# Etiological Spectrum and Maternal Peripartum Hematologic Outcomes of Thrombocytopenia in Pregnancy: A Retrospective Cohort Study

**DOI:** 10.3390/medicina62040771

**Published:** 2026-04-16

**Authors:** Bilge Erbey, Cemal Reşat Atalay, Sait Erbey

**Affiliations:** 1 Department of Obstetrics and Gynecology, Ankara Numune Training and Research Hospital, University of Health Sciences, Ankara 06010, Turkey; atalaycemal@yahoo.com.tr; 2 Department of Obstetrics and Gynecology, Ankara Etlik Şehir Hastanesi, Ankara 06170, Turkey; saiterbey@gmail.com

**Keywords:** thrombocytopenia, pregnancy, gestational thrombocytopenia, immune thrombocytopenia, mode of delivery, HELLP syndrome, platelet transfusion, peripartum hemorrhage

## Abstract

*Background and Objectives*: Thrombocytopenia complicates 6.6–11.6% of pregnancies. While gestational thrombocytopenia (GT) is usually benign, etiologies such as immune thrombocytopenia (ITP), preeclampsia, and HELLP syndrome require individualized management. This study aimed to characterize the etiological spectrum, maternal peripartum hematologic outcomes, blood product utilization, and mode of delivery in a tertiary-center cohort of thrombocytopenic pregnancies and to assess whether platelet count should influence delivery mode decisions. *Materials and Methods*: This retrospective cohort study included 137 thrombocytopenic pregnant women at a tertiary center (2010–2019), categorized by etiology and severity. Peripartum hemoglobin, hematocrit, and platelet counts were compared between delivery groups. Blood product utilization was recorded and analyzed using *t*-test, ANOVA, chi-square, Fisher’s exact, and Fisher–Freeman–Halton tests; binary logistic regression was used for multivariable analysis. *Results*: GT (43.1%) and ITP (32.1%) were the most prevalent diagnoses; cesarean delivery rate was 52.6%. Postpartum Hb was higher in the vaginal delivery group (10.24 ± 1.28 vs. 9.80 ± 1.26 g/dL; *p* = 0.003), while platelet counts were paradoxically lower (*p* = 0.039). Platelet transfusion rates did not differ significantly between delivery modes (23.1% vs. 27.8%; *p* = 0.621). Severe thrombocytopenia required platelet transfusion in 92.6% of cases versus 11.6% (moderate) and 0% (mild) (*p* < 0.001). RBC transfusion was highest in gestational hypertensive disease (41.2%) versus GT (5.1%) and ITP (2.3%) (*p* < 0.001). General anesthesia was used in 75% of cesarean cases. *Conclusions*: Delivery mode in thrombocytopenic pregnancies should be guided by obstetric indications, not platelet count alone. Although postpartum platelet counts declined more steeply after vaginal delivery, this did not increase transfusion requirements. Gestational hypertensive disorders carried the greatest hemorrhagic burden, highlighting the need for etiology-specific multidisciplinary planning. The high general anesthesia rate warrants prospective institutional audit of anesthetic decision-making protocols to determine adherence to current neuraxial anesthesia thresholds. This study is limited to maternal peripartum hematologic outcomes; neonatal outcomes were not captured and should be addressed in future prospective research.

## 1. Introduction

Thrombocytopenia, defined as a platelet count below 150 × 10^9^/L, complicates 6.6–11.6% of pregnancies and represents the second most common hematologic abnormality in this population after anemia [1,2]. The differential diagnosis is broad, encompassing conditions unique to pregnancy—gestational thrombocytopenia, preeclampsia, HELLP (hemolysis, elevated liver enzymes, low platelets) syndrome—as well as systemic disorders coinciding with pregnancy, such as immune thrombocytopenia (ITP), thrombotic thrombocytopenic purpura (TTP), hemolytic uremic syndrome (HUS), and drug-induced thrombocytopenia [3,4]. Accurate etiological differentiation is essential, as management pathways diverge substantially across these conditions.

The majority of cases (65–80%) are attributable to gestational thrombocytopenia, a benign, self-limiting condition that resolves postpartum without maternal or neonatal sequelae [2,5]. ITP, the most frequent cause of isolated thrombocytopenia in the first two trimesters, accounts for 5–15% of cases and requires a different management approach, including potential pharmacological intervention and neonatal surveillance [6,7]. At the severe end of the spectrum, preeclampsia-associated thrombocytopenia and HELLP syndrome may precipitate life-threatening hemorrhage, requiring urgent delivery [8,9].

A major unresolved clinical question is whether the mode of delivery should be influenced by platelet count in thrombocytopenic pregnancies. Current guidelines from the American College of Obstetricians and Gynecologists (ACOG) and the American Society of Hematology (ASH) explicitly advise against performing cesarean delivery solely on the basis of maternal thrombocytopenia [7,10]. Nevertheless, platelet thresholds continue to guide anesthetic decisions: the 2021 Society for Obstetric Anesthesia and Perinatology (SOAP) consensus statement endorses neuraxial anesthesia when platelet counts are ≥70 × 10^9^/L in stable patients [11]. Real-world adherence to these evidence-based thresholds, and their downstream impact on maternal peripartum hematologic outcomes, remains incompletely characterized.

Because randomized trials in this population are both ethically and logistically constrained, large etiology-stratified retrospective series from tertiary centers remain essential contributors to the evidence base. We therefore conducted a nine-year, single-center retrospective cohort study of 137 thrombocytopenic pregnancies. We hypothesized that, after accounting for etiological subgroup and obstetric indications, mode of delivery would not predict peripartum hematologic outcomes or blood product requirements. The specific objectives were to characterize the etiological spectrum, peripartum hematologic trajectories, blood product utilization, and delivery mode distribution in this cohort and to evaluate whether thrombocytopenia severity or etiology should influence anesthetic and delivery planning.

## 2. Materials and Methods

### 2.1. Study Design and Setting

This retrospective cohort study was conducted at the Department of Obstetrics and Gynecology of Ankara Numune Training and Research Hospital, a tertiary referral center affiliated with the University of Health Sciences, Ankara, Turkey. The study period was January 2010 through January 2019. Ethics committee approval was obtained retrospectively prior to data analysis, as is standard for retrospective medical record reviews in Turkey (Approval No.: 20190127, date: 12 January 2019). All patient data were de-identified prior to analysis. Due to the retrospective nature of this study, individual patient informed consent was waived in accordance with applicable national regulations.

### 2.2. Participants

Eligible participants were pregnant women with confirmed thrombocytopenia (platelet count <150 × 10^9^/L on at least two consecutive measurements taken ≥48 h apart) who delivered at our institution during the study period. Only patients with persistent thrombocytopenia (confirmed on both measurements) were included; cases in which the platelet count normalized between the two measurements were excluded as transient thrombocytopenia. Patients were identified through a systematic review of the obstetric database. Exclusion criteria were as follows: (i) loss to follow-up before delivery (*n* = 7); (ii) delivery at another institution (*n* = 6); (iii) incomplete laboratory records at either the predelivery or 24 h postdelivery time point; (iv) confirmed diagnosis of a systemic illness known to independently cause thrombocytopenia (e.g., systemic lupus erythematosus, antiphospholipid syndrome, chronic liver disease) unless the thrombocytopenia could be attributed to a co-existing pregnancy-related etiology by multidisciplinary consensus. Of 150 initially identified patients, 137 were included in the final analysis.

### 2.3. Etiological Classification

All diagnoses were assigned by the responsible obstetrics team in consultation with hematology, applying the following established criteria. In cases where features of more than one condition were present simultaneously (e.g., preeclampsia with coincident ITP-like thrombocytopenia), the diagnosis was assigned based on the predominant and most clinically acute etiology by multidisciplinary consensus; no patient received a dual primary diagnosis.

**Gestational thrombocytopenia (GT):** Isolated, asymptomatic thrombocytopenia first detected in the second or third trimester, in the absence of a prior history of thrombocytopenia, a systemic disease, or medications known to cause thrombocytopenia, with documented spontaneous resolution within 12 weeks postpartum [5,12].

**Immune thrombocytopenia (ITP):** Isolated thrombocytopenia (platelet count <100 × 10^9^/L) after exclusion of other identifiable causes, including GT, hypertensive disorders, infection, and drug effects, classified per ASH 2019 and BCSH 2003 guidelines [7,13].

**Preeclampsia/Eclampsia:** Defined as new-onset hypertension (≥140/90 mmHg) with proteinuria or evidence of end-organ damage after 20 weeks of gestation; eclampsia was defined as seizures superimposed on preeclampsia, per ACOG criteria [10].

**HELLP syndrome:** Diagnosed using the Tennessee Classification System: microangiopathic hemolytic anemia, AST ≥ 70 U/L, LDH > 600 U/L, and platelets < 100 × 10^9^/L [8].

**Pseudothrombocytopenia:** Confirmed by peripheral blood smear review demonstrating platelet clumping and/or normalization in a citrate-anticoagulated sample [14]. Citrate-anticoagulated repeat testing was performed in all cases where pseudothrombocytopenia was clinically suspected based on the absence of bleeding symptoms, a discrepancy between automated platelet count and clinical findings, or the presence of clumping on the initial smear. These patients were retained as a reference subgroup. A sensitivity analysis excluding this subgroup (*n* = 136) showed results consistent with the main analysis: RBC transfusion 3.1% vs. 13.9% by delivery mode (*p* = 0.034); platelet transfusion 21.9% vs. 27.8% (*p* = 0.552); postdelivery platelet counts lower after vaginal delivery (105,656 ± 22,777 vs. 114,167 ± 25,200 × 10^9^/L; *p* = 0.042); and platelet transfusion rates by severity 0% (mild), 11.6% (moderate), and 92.3% (severe) (*p* < 0.001). These results confirm that pseudothrombocytopenia cases did not materially affect the study conclusions.

Additional diagnoses (lymphoproliferative disease, thalassemia trait, aplastic anemia, maternal malignancy) were assigned by relevant subspecialty teams. Among lymphoproliferative cases, three had Hodgkin lymphoma and one non-Hodgkin lymphoma; the single malignancy case involved cervical carcinoma managed conservatively until delivery. These cases are presented descriptively.

Thrombocytopenia severity was categorized as mild (100–150 × 10^9^/L), moderate (50–100 × 10^9^/L), and severe (<50 × 10^9^/L), per Bergmann and Rath [3].

### 2.4. Outcomes and Data Collection

Primary outcomes were peripartum hematologic trajectories (predelivery and 24 h postdelivery Hb, Hct, and platelet counts) and blood product utilization, assessed across etiological subgroups and delivery modes. Secondary outcomes included mode of delivery, anesthesia type, and cesarean indication. Platelet transfusion was administered according to institutional protocol at thresholds of <50 × 10^9^/L prior to cesarean delivery, <80 × 10^9^/L when neuraxial anesthesia was planned, and <20 × 10^9^/L in the absence of active bleeding for vaginal delivery; individualized decisions at lower or higher thresholds were made at the discretion of the attending hematologist in consultation with obstetrics. RBC transfusion was guided by clinical symptoms and a hemoglobin threshold of <7 g/dL, or <8 g/dL in the presence of cardiovascular compromise. Data were extracted from electronic medical records by two independent reviewers; discrepancies were resolved by consensus with a third reviewer.

### 2.5. Statistical Analysis

Continuous variables were tested for normality (Shapiro–Wilk) and expressed as mean ± SD or median (IQR). Between-group comparisons used Student’s *t*-test, one-way ANOVA with Tukey post hoc correction, or Mann–Whitney U/Kruskal–Wallis tests as appropriate. Within-subject predelivery-to-postdelivery changes were evaluated by paired *t*-test. Categorical variables were compared using chi-square or Fisher’s exact test. For the univariate comparison of RBC transfusion by delivery mode, Fisher’s exact test was used given sparse cell counts. Binary logistic regression was performed to assess the independent association of delivery mode with (a) RBC transfusion and (b) platelet transfusion, after adjustment for etiological group (dummy-coded: ITP, gestational hypertensive disease, and other diagnoses, with GT as reference), thrombocytopenia severity (ordinal: 0 = mild, 1 = moderate, 2 = severe), and gestational age at delivery (standardized). Odds ratios (OR) with 95% confidence intervals (CI) were calculated using the Wald method. A *p*-value < 0.05 was considered statistically significant. A sensitivity analysis was performed excluding pseudothrombocytopenia cases (*n* = 136). All analyses used SPSS version 22.0 (IBM Corp., Armonk, NY, USA).

## 3. Results

### 3.1. Patient Characteristics

A total of 137 patients were included. Mean age was 29.3 ± 5.8 years; mean gravidity 2.37 ± 1.27; mean parity 0.98 ± 0.88 (Table 1). Gestational age at delivery ranged from 25 to 41 weeks (mean 36.55 ± 3.37 weeks). Sixty-five patients (47.4%) delivered vaginally and 72 (52.6%) by cesarean section. The cesarean group had a significantly lower mean gestational age at delivery compared to the vaginal delivery group (35.85 ± 3.92 vs. 37.32 ± 2.24 weeks; *p* = 0.009), largely attributable to the over-representation of gestational hypertensive disease patients in the cesarean group.

### 3.2. Etiological Distribution

GT was the most common diagnosis (59/137; 43.1%), followed by ITP (44/137; 32.1%), preeclampsia (11/137; 8.0%), pseudothrombocytopenia (9/137; 6.6%), lymphoproliferative disease (4/137; 2.9%), eclampsia (3/137; 2.2%), HELLP syndrome (3/137; 2.2%), thalassemia trait (2/137; 1.5%), aplastic anemia (1/137; 0.7%), and maternal malignancy (1/137; 0.7%) (Table 2).

### 3.3. Mode of Delivery and Anesthesia

Among 72 cesarean deliveries, the most common indications were previous cesarean section (43.1%), fetal distress (23.6%), eclampsia (11.1%), placental abruption (5.6%), transverse lie (5.6%), breech presentation (4.2%), HELLP syndrome (4.2%), and macrosomia (1.4%). General anesthesia was used in 54 (75.0%) cesarean cases and spinal anesthesia in 18 (25.0%). The distribution of diagnoses across delivery modes differed significantly (*p* = 0.001): GT and ITP were more common in the vaginal delivery group, whereas gestational hypertensive disorders were disproportionately represented in the cesarean group (Table 3). The type of anesthesia also varied significantly by diagnosis (*p* = 0.010).

### 3.4. Peripartum Hematologic Outcomes by Delivery Mode

Predelivery Hb and Hct were similar between groups. Postdelivery Hb was significantly higher following vaginal delivery (10.24 ± 1.28 vs. 9.80 ± 1.26 g/dL; *p* = 0.003; Cohen’s d = 0.35) as was Hct (30.31 ± 3.72 vs. 29.31 ± 3.65%; *p* = 0.008; d = 0.27); however, this difference is heavily confounded by case-mix and should not be interpreted as a direct effect of delivery mode. Conversely, postdelivery platelet counts were significantly lower in the vaginal group (105,600 ± 24,100 vs. 113,800 ± 22,900 ×10^9^/L; *p* = 0.039; d = 0.35) despite comparable predelivery levels (Table 4); all effect sizes were modest.

RBC transfusion was required in 2/65 (3.1%) vaginal and 10/72 (13.9%) cesarean patients (*p* = 0.033; OR 5.08, 95% CI 1.07–24.1, Fisher’s exact test). Platelet transfusion rates did not differ significantly: 15/65 (23.1%) vaginal vs. 20/72 (27.8%) cesarean (*p* = 0.621; OR 1.28, 95% CI 0.58–2.81) (Table 4), indicating that thrombocytopenia severity—not delivery mode—drives platelet transfusion need.

### 3.5. Multivariable Logistic Regression: Predictors of Transfusion

Binary logistic regression models adjusting for etiological group, thrombocytopenia severity, and gestational age are presented in Table 5. After multivariable adjustment, delivery mode was not independently associated with RBC transfusion (OR 1.74, 95% CI 0.28–10.8; *p* = 0.55) or platelet transfusion (OR 0.90, 95% CI 0.22–3.65; *p* = 0.88). Thrombocytopenia severity was the sole independent predictor of platelet transfusion need (OR 108.7, 95% CI 20.7–570.8; *p* < 0.001); the wide confidence interval reflects the small number of events (*n* = 35) relative to the six predictors in the model and should be interpreted with caution. Lower gestational age at delivery was the only independent predictor of RBC transfusion (OR 0.52 per SD increase in gestational age, 95% CI 0.28–0.99; *p* = 0.046), consistent with preterm deliveries carrying greater hemorrhagic risk in this cohort.

### 3.6. Outcomes by Severity of Thrombocytopenia

Among all 137 patients, thrombocytopenia was classified as mild in 22 (16.1%), moderate in 88 (64.2%), and severe in 27 (19.7%). The distribution of severity varied significantly by diagnosis (*p* = 0.029): ITP had the highest rate of severe thrombocytopenia (29.5%) compared to pseudothrombocytopenia (0%) and GT (11.9%). Platelet transfusion was required in 25/27 (92.6%) patients with severe, 10/88 (11.4%) with moderate, and 0/22 (0%) with mild thrombocytopenia (*p* < 0.001). RBC transfusion rates did not differ significantly across severity categories (*p* = 0.124).

### 3.7. Maternal Peripartum Hematologic Outcomes by Etiological Subgroup

Predelivery platelet counts differed significantly across diagnostic groups (one-way ANOVA F = 4.731, *p* = 0.004). ITP patients had the lowest predelivery platelet counts (63,341 ± 24,324 × 10^9^/L), while pseudothrombocytopenia patients had the highest (90,333 ± 23,869 × 10^9^/L). Within all major diagnostic groups, postdelivery platelet counts were significantly higher than predelivery counts (all *p* < 0.001 by paired *t*-test), indicating postpartum recovery regardless of etiology. Postdelivery platelet counts also differed significantly across groups (one-way ANOVA F = 3.902, *p* = 0.011). Post hoc analysis showed that ITP patients had lower postdelivery platelet counts compared to gestational hypertensive disease patients (*p* = 0.008, Bonferroni-corrected), with a trend toward lower counts versus GT (*p* = 0.075), reflecting persistent autoimmune platelet destruction (Table 6).

RBC transfusion requirements differed significantly by diagnosis (*p* < 0.001): 7/17 (41.2%) of gestational hypertensive disease patients required RBC transfusion versus 3/59 (5.1%) GT, 1/44 (2.3%) ITP, and 0/9 (0%) pseudothrombocytopenia patients. Platelet transfusion rates also differed (*p* = 0.009): pseudothrombocytopenia (0%), GT (16.9%), ITP (34.1%), and gestational hypertensive disease (47.1%) (Table 7).

## 4. Discussion

Three clinically relevant findings emerged from this nine-year cohort. First, delivery mode was not associated with platelet transfusion requirements in either univariate or multivariable analysis, consistent with guideline recommendations against cesarean delivery solely for thrombocytopenia. Second, gestational hypertensive disorders disproportionately drove red cell transfusion, emphasizing etiology over severity in hemorrhagic risk. Third, general anesthesia was used in 75% of cesarean cases, a rate that warrants prospective institutional audit.

The etiological distribution—GT (43.1%), ITP (32.1%), gestational hypertensive disease (12.4%), pseudothrombocytopenia (6.6%), and rare causes (7.3%)—broadly reflects established epidemiology [2,5,12]. The higher ITP proportion compared to population-based series (5–15%) likely reflects tertiary referral bias, with complex ITP cases requiring hematology co-management disproportionately referred to our center; this must be considered when generalizing these findings.

Postpartum Hb and Hct were observed to be higher after vaginal delivery; however, this difference is strongly limited by case-mix and confounding by indication and should not be interpreted as reflecting greater blood loss with cesarean section [15]. This difference largely reflects severe confounding by indication: gestational hypertensive disease patients—who have the highest intrinsic hemorrhagic risk—were almost exclusively in the cesarean group (94.1%), and they accounted for the vast majority of RBC transfusions. Consequently, observed differences in Hb, Hct, and RBC transfusion rates between delivery mode groups primarily reflect the more severe clinical profile of cesarean patients rather than the true effect of delivery mode itself. Notably, although postdelivery Hb and Hct differed significantly between groups (*p* = 0.003; *p* = 0.008), the absolute changes (ΔHb, ΔHct) did not (*p* = 0.168; *p* = 0.397), because both groups lost similar absolute amounts from slightly different baselines—a complementary finding that does not imply greater hemorrhage with cesarean delivery.

Postpartum platelet counts were significantly lower after vaginal compared to cesarean delivery (*p* = 0.039), despite comparable predelivery levels. Possible mechanisms include greater hemostatic activation during labor and placental separation, oxytocin-mediated platelet aggregation, and more controlled surgical hemostasis limiting platelet demand in cesarean delivery [2,16]. Importantly, this platelet decline did not translate into differences in platelet transfusion rates (vaginal 23.1% vs. cesarean 27.8%; *p* = 0.621), confirming that platelet count trajectory alone should not drive delivery mode selection. RBC transfusion rates showed a statistically significant difference in univariate analysis (vaginal 3.1% vs. cesarean 13.9%; OR 5.08, 95% CI 1.07–24.1; *p* = 0.033, Fisher’s exact test); however, in multivariable analysis adjusting for etiology, thrombocytopenia severity, and gestational age, delivery mode was no longer a significant predictor of RBC transfusion (OR 1.74, 95% CI 0.28–10.8; *p* = 0.55), suggesting that this association is primarily driven by confounding by indication rather than by delivery mode itself.

These findings support current ACOG and ASH recommendations that cesarean delivery should not be performed solely on the basis of maternal thrombocytopenia [7,10]. Delivery mode decisions should be driven by obstetric indications, with platelet management addressed in parallel. The high rate of previous cesarean section (43.1%) as the leading indication reflects Turkey’s nationally elevated cesarean rate [17,18].

The high rate of general anesthesia in our cesarean cohort (75.0%) warrants careful consideration. The 2021 SOAP consensus recommends neuraxial anesthesia as safe for stable platelet counts ≥70 × 10^9^/L [11]. This high rate likely reflects diagnostic uncertainty between GT and ITP rather than truly contraindicated platelet counts; in the absence of a reliable discriminating test, clinicians may understandably default to general anesthesia [5,12]. Because individual platelet counts at the time of the anesthetic decision were not systematically documented, the present data do not allow assessment of whether individual anesthetic decisions conformed to SOAP recommendations. This finding should therefore be interpreted as an organizational signal warranting prospective institutional audit, rather than as evidence of suboptimal clinical practice.

The severity-based analysis confirms that severe thrombocytopenia (<50 × 10^9^/L) is a robust predictor of platelet transfusion need (92.6%), consistent with guideline-recommended thresholds: platelet count > 50 × 10^9^/L for surgical delivery and >70–80 × 10^9^/L for neuraxial anesthesia [7,10,11]. This underscores the importance of severity classification at admission to guide resource planning, particularly in low-resource or high-volume settings.

The markedly elevated RBC transfusion rate in gestational hypertensive disease patients (41.2% vs. 0–5.1% in other groups) underscores the hemorrhagic burden of this subgroup. HELLP syndrome involves consumptive coagulopathy superimposed on hemolytic anemia, with platelet nadir typically occurring 24–48 h postdelivery [8,19]. The 24 h postdelivery measurement window used in this study may therefore not capture the true platelet nadir in HELLP syndrome; postdelivery platelet values for this subgroup should be interpreted as a partial, potentially underestimated representation of the postpartum hematologic trajectory. The pooled gestational hypertensive disease group (n = 17) consistently showed the highest transfusion burden, supporting proactive multidisciplinary peripartum planning in these patients.

The higher platelet transfusion rate (34.1%) and lower postdelivery platelet counts in ITP versus GT reflect persistent autoimmune platelet destruction postpartum [6,7]. Bleeding complications in pregnant women with ITP, while generally manageable, can be clinically significant and have been documented in large meta-analytic series [20]. Current guidelines recommend treatment when counts fall below 20–30 × 10^9^/L or in the presence of bleeding [21,22]. First-line corticosteroids (with or without IVIG) achieve response in 70–80% of patients; thrombopoietin receptor agonists are increasingly used for refractory cases in pregnancy [23]. Neonatal thrombocytopenia from transplacental maternal IgG occurs in up to 10–15% of neonates [6,24]; however, neonatal outcomes were not captured in this study due to the absence of a co-located NICU, representing the most significant gap in this dataset and limiting the comprehensiveness of the clinical picture presented.

### Strengths and Limitations

Strengths include the large single-institution sample, decade-long follow-up, systematic etiological subgrouping, and inclusion of both hematologic outcomes and transfusion data. Limitations include the following. First, the retrospective design precludes causal inference and cannot fully disentangle confounding by indication. Although binary logistic regression models were added for the primary transfusion outcomes (Table 5), the remaining analyses are univariate; the absence of multivariable adjustment for Hb, Hct, and platelet outcomes means that observed associations between delivery mode and these hematologic parameters cannot be causally attributed to delivery mode per se, as they are heavily influenced by the disproportionate representation of gestational hypertensive disease in the cesarean group. Furthermore, several clinically relevant confounders—including pre-existing bleeding history, coagulation parameters (e.g., fibrinogen, PT/aPTT), and inter-clinician variability in management decisions—were not captured in the dataset and could not be included in multivariable models, which may have influenced both transfusion and anesthetic outcomes. Second, no formal power calculation was performed; accordingly, non-significant findings in small subgroup analyses (e.g., HELLP, aplastic anemia) cannot be interpreted as evidence of no effect, and this study may be underpowered to detect meaningful differences in these subgroups. Third, the potential for etiological misclassification exists, particularly between gestational thrombocytopenia and early ITP, and between preeclampsia-associated thrombocytopenia and HELLP syndrome with overlapping features; consensus-based multidisciplinary classification was used to minimize this risk but cannot be fully eliminated in a retrospective design. Fourth, the 24 h postdelivery measurement time point may not capture the true platelet nadir, particularly in HELLP syndrome where the nadir typically occurs 24–48 h postdelivery, but also potentially in other conditions with delayed postpartum platelet consumption; postdelivery platelet values should therefore be interpreted as a partial representation of the postpartum hematologic trajectory. Fifth, while institutional transfusion and anesthesia thresholds were predefined, the retrospective design does not permit verification of whether all individual management decisions strictly adhered to protocol; clinician-level variability in the application of thresholds cannot be excluded and may have contributed to heterogeneity in observed transfusion and anesthesia rates. Sixth, neonatal outcomes—including neonatal thrombocytopenia from transplacental IgG in ITP, neonatal bleeding complications, and other perinatal morbidities—were unavailable due to the absence of a co-located NICU; this study should therefore be interpreted strictly as an analysis of maternal peripartum hematologic management rather than a comprehensive assessment of peripartum outcomes, and the clinical relevance of the findings is accordingly limited to the maternal domain. The high rate of previous cesarean sections as a cesarean indication (43.1%) reflects Turkey’s nationally elevated cesarean rate rather than thrombocytopenia-driven decisions. Future prospective multicenter studies with neonatal data linkage, standardized transfusion protocols, formal power calculations, and comprehensive multivariable modeling are needed.

## 5. Conclusions

This nine-year tertiary center cohort demonstrates that thrombocytopenia in pregnancy encompasses a clinically diverse spectrum, with gestational thrombocytopenia and ITP collectively accounting for three-quarters of cases. This study is explicitly limited to maternal peripartum hematologic outcomes; neonatal outcomes were not captured and should be addressed in future research. While postpartum hemoglobin and hematocrit were observed to be higher following vaginal delivery—an association strongly limited by case-mix and confounding by indication rather than reflecting a causal effect of delivery mode—postpartum platelet counts paradoxically declined more after vaginal delivery. These between-group differences are heavily confounded by the disproportionate concentration of gestational hypertensive disease patients in the cesarean group and should not be interpreted as direct effects of delivery mode. Critically, this platelet differential did not translate into differences in platelet transfusion requirements, consistent with recommendations that mode of delivery should be guided by obstetric indications rather than platelet count alone. Multivariable logistic regression (Table 5) confirms that delivery mode is not an independent predictor of transfusion need after adjustment for etiology, severity, and gestational age. Nevertheless, platelet count and etiology should be regarded as equally essential parameters in multidisciplinary peripartum planning, particularly for anesthesia decisions, where severe thrombocytopenia (<50 × 10^9^/L) mandated transfusion in 92.6% of cases and directly constrained access to neuraxial anesthesia. Gestational hypertensive disorders carried the highest peripartum hemorrhagic risk, with RBC transfusion needed in 41.2% of affected patients, underscoring the importance of etiology-specific risk stratification and multidisciplinary planning. Severe thrombocytopenia reliably predicts platelet transfusion need and warrants proactive hemostatic preparation regardless of delivery mode.

## Figures and Tables

**Table 1:** Baseline demographic and obstetric characteristics (n = 137).

Variable	n/Mean ± SD/Median	Range/IQR
Age (years)	29.3 ± 5.8	18–44
Gravidity	2.37 ± 1.27	1–9
Parity	0.98 ± 0.88	0–4
Spontaneous abortions	0.40 ± 0.81	0–6
Gestational age at delivery (weeks)	36.55 ± 3.37	25–41
Vaginal delivery, *n* (%)	65 (47.4%)	—
Cesarean delivery, *n* (%)	72 (52.6%)	—
GA at delivery—vaginal (weeks)	37.32 ± 2.24	*p* = 0.009 *
GA at delivery—cesarean (weeks)	35.85 ± 3.92	

GA = gestational age; * Student’s *t*-test; *p* < 0.05.

**Table 2:** Etiological distribution of thrombocytopenia (n = 137).

Diagnosis	*n*	%
Gestational thrombocytopenia	59	43.1
Immune thrombocytopenia (ITP)	44	32.1
Preeclampsia	11	8
Pseudothrombocytopenia	9	6.6
Lymphoproliferative disease	4	2.9
Eclampsia	3	2.2
HELLP syndrome	3	2.2
Thalassemia trait	2	1.5
Aplastic anemia	1	0.7
Maternal malignancy	1	0.7
Total	137	100

HELLP = hemolysis, elevated liver enzymes, low platelets.

**Table 3:** Distribution of diagnoses by mode of delivery.

Diagnosis	Vaginal *n* (%)	Cesarean *n* (%)	*p*
Gestational thrombocytopenia	34 (57.6%)	25 (42.4%)	<0.001 *
Immune thrombocytopenia	25 (56.8%)	19 (43.2%)	
Pseudothrombocytopenia	4 (44.4%)	5 (55.6%)	
Gestational hypertensive disease †	1 (5.9%)	16 (94.1%)	
Other ‡	1 (12.5%)	7 (87.5%)	

* Fisher–Freeman–Halton test. † Includes preeclampsia, eclampsia, HELLP. ‡ Lymphoproliferative disease, thalassemia trait, aplastic anemia, maternal malignancy. Percentages are row-based (proportion of each diagnostic group delivered by each mode).

**Table 4:** Peripartum hematologic parameters and transfusion rates by delivery mode (mean ± SD).

Parameter	Vaginal (*n* = 65)	Cesarean (*n* = 72)	*p*-Value
Predelivery Hb (g/dL)	11.48 ± 1.41	11.27 ± 1.39	0.378
Postdelivery Hb (g/dL)	10.24 ± 1.28	9.80 ± 1.26	0.003 *
ΔHb predelivery–postdelivery (g/dL)	1.24 ± 1.01	1.47 ± 0.95	0.168
Predelivery Hct (%)	33.85 ± 4.13	33.22 ± 4.10	0.362
Postdelivery Hct (%)	30.31 ± 3.72	29.31 ± 3.65	0.008 *
ΔHct predelivery–postdelivery (%)	3.54 ± 3.12	3.91 ± 2.78	0.397
Predelivery platelet (×10^9^/L)	74,210 ± 24,300	72,400 ± 25,100	0.657
Postdelivery platelet (×10^9^/L)	105,600 ± 24,100	113,800 ± 22,900	0.039 *
RBC transfusion, *n* (%)	2 (3.1%)	10 (13.9%)	0.033 *
Platelet transfusion, *n* (%)	15 (23.1%)	20 (27.8%)	0.621

Hb = hemoglobin; Hct = hematocrit; RBC = red blood cell; Δ = change from predelivery to 24 h postdelivery. * *p* < 0.05. Continuous variables: Student’s *t*-test (normally distributed) or Mann–Whitney U test (non-normally distributed), per Shapiro–Wilk testing. Fisher’s exact test for RBC transfusion; chi-square test for platelet transfusion.

**Table 5:** Multivariable binary logistic regression: independent predictors of RBC and platelet transfusion (n = 137).

Predictor	OR (RBC)	95% CI	*p*	OR (PLT)	95% CI	*p*
Delivery mode (CS vs. vaginal)	1.74	0.28–10.8	0.55	0.90	0.22–3.65	0.88
Severity (ordinal: mild = 0, moderate = 1, severe = 2)	2.25	0.75–6.72	0.15	**108.72**	**20.71–570.8**	<0.001 *
Gestational age (per SD increase)	**0.52**	**0.28–0.99**	0.046 *	0.87	0.45–1.69	0.69
ITP vs. GT	0.49	0.04–5.44	0.56	1.36	0.31–6.01	0.69
Gestational HT vs. GT	4.50	0.72–28.2	0.11	5.72	0.70–47.0	0.10
Other diagnoses vs. GT	1.01	0.08–12.2	0.99	0.44	0.03–7.23	0.57

GT = gestational thrombocytopenia; ITP = immune thrombocytopenia; HT = hypertensive disease; CS = cesarean section; OR = odds ratio; CI = confidence interval. Reference category for etiology: GT. * *p* < 0.05. Bold values in the regression table indicate statistically significant predictors (*p* < 0.05).

**Table 6:** Pre- and postdelivery platelet counts by diagnostic group (×10^9^/L, mean ± SD).

Group	Predelivery Platelet	Postdelivery Platelet	*p* (Within) †	^1^ *p* (Between)
GT (n = 59)	78,983 ± 22,775	113,102 ± 25,431	<0.001 *	0.004 *
ITP (n = 44)	63,341 ± 24,324	100,977 ± 21,761	<0.001 *	
Pseudothrombocytopenia (*n* = 9)	90,333 ± 23,869	127,333 ± 27,404	<0.001 *	
Gest. hypertensive disease (*n* = 17)	69,824 ± 27,263	115,706 ± 19,684	<0.001 *	

GT = gestational thrombocytopenia; † Paired samples *t*-test (within-group pre-to-post comparison); ^1^ One-way ANOVA for predelivery and postdelivery platelet counts across all four diagnostic groups. Predelivery: one-way ANOVA F = 4.731, *p* = 0.004; post hoc Bonferroni: ITP vs. GT *p* = 0.007. Postdelivery: one-way ANOVA F = 3.902, *p* = 0.011; post hoc Bonferroni: ITP vs. GT *p* = 0.075 (trend), ITP vs. gestational hypertensive disease *p* = 0.008. * *p* < 0.05.

**Table 7:** Blood product utilization by diagnostic group.

Diagnosis	RBC Transfusion *n* (%)	Platelet Transfusion *n* (%)	*p* (RBC)	*p* (PLT)
GT (*n* = 59)	3 (5.1%)	10 (16.9%)	<0.001 *	0.009 *
ITP (*n* = 44)	1 (2.3%)	15 (34.1%)		
Pseudothrombocytopenia (*n* = 9)	0 (0%)	0 (0%)		
Gest. hypertensive disease (*n* = 17)	7 (41.2%)	8 (47.1%)		

GT = gestational thrombocytopenia; RBC = red blood cell; PLT = platelet transfusion. * Fisher–Freeman–Halton test; *p* < 0.05.

## Data Availability

The datasets used and analyzed during the current study are available from the corresponding author upon reasonable request, subject to institutional data governance regulations.
